# Rural Farmers’ Cognition and Climate Change Adaptation Impact on Cash Crop Productivity: Evidence from a Recent Study

**DOI:** 10.3390/ijerph191912556

**Published:** 2022-10-01

**Authors:** Nawab Khan, Jiliang Ma, Hazem S. Kassem, Rizwan Kazim, Ram L. Ray, Muhammad Ihtisham, Shemei Zhang

**Affiliations:** 1 College of Management, Sichuan Agricultural University, Chengdu 611130, China; 2 Institute of Agricultural Economics and Development, Chinese Academy of Agricultural Sciences, Beijing 100081, China; 3 Department of Agricultural Extension and Rural Society, King Saud University, Riyadh 11451, Saudi Arabia; 4 College of Agriculture and Human Sciences, Prairie View A&M University, Prairie View, TX 77446, USA; 5 School of Agriculture, Forestry and Food Engineering, Yibin University, Yibin 644000, China; 6 College of Horticulture and Forestry, Huazhong Agricultural University, Wuhan 430070, China

**Keywords:** cognition, climate change, adaptation, cash crop productivity, agriculture, Pakistan

## Abstract

The world faces a once-in-a-century transformation due to the COVID-19 pandemic, adversely affecting farmers’ employment, production practices, and livelihood resilience. Meanwhile, climate change (CC) is a crucial issue limiting agricultural production worldwide. Farmers’ lives, severely affected by extreme weather conditions, are resulting in the reduced production of major economic crops. The CC has drastically influenced the major agricultural sectors of Pakistan, leading to a significant decline in farmers’ living standards and the overall economy. Climate-smart and eco-friendly agricultural practices can mitigate greenhouse gas emissions and ameliorate agricultural productivity under extreme environmental conditions. This paper highlights farmers’ autonomous CC adaptation strategies and their influence on cash crop (maize for this study) yield under prevailing circumstances. The current study used a simultaneous equation model to examine the different adaptation impacts on adapters and non-adapters. The survey results of 498 maize farmers in rural Pakistan revealed that growers were aware of the recent CC and had taken adequate adaptive measures to acclimatize to CC. Farmers’ arable land area, awareness level, and information accessibility to CC are the most crucial factors that impart a significant role in their adaptation judgments. However, most growers have inadequate adaptation strategies, including improved irrigation and the utilization of extensive fertilizers and pesticides. Using a simultaneous equation model of endogenous switching regression, the study found that farmers not adapted to CC were negatively affecting maize productivity. Therefore, this study suggests that policymakers pay attention to the countermeasures farmers have not taken to mitigate the impact of CC. In addition, policymakers should deliver appropriate adaptation strategies to assist growers in coping with climate-related natural hazards and ensure farmers’ livelihood security, rural revitalization, and sustainable agricultural development.

## 1. Introduction

Under the COVID-19 pandemic, the world faces a once-in-a-century transformation that substantially affects farmers’ employment, agricultural production practices, and livelihoods. While climate change and anthropogenic activities threaten agricultural sustainability, the ongoing COVID-19 pandemic significantly impacted agricultural activities [1,2,3,4,5]. Several studies reported considerable direct and indirect impacts of the ongoing pandemic on agriculture due to restrictions in labor for planting, seeding, harvesting, processing, production, and marketing, through delivery and logistical constraints and limitations to access critical farm inputs during the lockdown [2,3,4,6,7,8,9]. For example, Gregorio and Ancog [10] reported a 3.11% (17.03 million tons) reduction in agricultural production in Southeast Asia due to a decline in farm labor. Simultaneously, climate change (CC) has gradually become a critical issue globally [11]. The CC and associated natural disasters are reshaping agricultural production and development patterns, thereby disturbing the resilience and sustainability of farmers’ livelihoods.

Global climate change is an undeniable fact and Pakistan is one of the countries with the most apparent destructive consequences. From 1999 to 2018, Pakistan suffered a total loss of US$3.79 million due to CC. Therefore, the long-term global climate risk index survey ranks Pakistan as the fifth most highly disturbed country globally [12,13]. In the last century, a temperature increase of 0.6 to 1.0 °C and increased precipitation from 18% to 32% has been observed, which may undermine the productivity of the agricultural sector in agriculture-dependent economies such as Pakistan [14]. Some reports predict that the situation will become more critical in the future, which will cause severe problems for Pakistan’s agriculture [15]. The climate-related issues impact both developed and developing countries. Still, in emerging nations, because of low adaptive capability, the effect is even more apparent and lethal [12,16,17]. Although mitigation efforts are the best way to meet the challenges of CC, it requires a lot of financial resources, effort, and time. In developing countries, such as Pakistan, adaption to varying climatic conditions is the best way to diminish the hazardous consequences of CC in the agricultural sector [18,19,20]. Pakistan’s economy is highly dependent on farming, which accounts for a massive share of the annual gross domestic product, up to 18.9% [21]. Although the agricultural sector is essential for Pakistan’s economy, it also faces several challenges. However, climate-related disasters, such as droughts and floods, are crucial issues [21,22].

The imminent risks associated with CC are obvious and real, but as far as the agricultural sector is concerned they are quite indefinite; hence, adaptation is not only an efficient technique but also limits the adverse effects of environmental hazards [23]. Almost every society is adaptable, but CC perception can play a significant role, and adaptability is closely related to education, resource acquisition, and awareness. However, small farmers in Pakistan are incompetent in obtaining these components. A larger proportion of the population (29.5%) lives below the poverty line, which undermines the ability of farmers to solve CC issues [12]. Therefore, for emerging nations, adaptation is challenging, where CC acquaintance is high and poverty and low farm-level adaptability further exacerbate this situation [19,24,25,26]. In addition to farmers’ low technical and financial capabilities, ineffective climate policies limit current support for CC adaptation [27]. Consequently, it is necessary to formulate a targeted adaptation policy to understand the factors that affect farmers’ cognition and adaptive response [28,29]. Although different adaptation measures adopted by growers are related to social, environmental, and economic aspects [30,31], the perception of CC is crucial. Hence, it is essential to investigate how growers understand CC and how they respond to it. In addition, the form and scope of adaptation approaches used for mitigation are critical to prospects [25,32]. Although extensive research has been conducted on farmers’ perception and adaptive behavior under CC, the decisive factor affecting adaptive behavior needs more investigation [12,19,26,33,34,35].

In Pakistan, research on CC issues is limited to CC impact and its predictions for specific crop production so far. Therefore, this study aims to fill the current research gaps in the agricultural field because maize is Pakistan’s major food crop. According to a labor force survey conducted by the bureau of statistics of Pakistan (2017–2018), 39% of the workforce is engaged in the agriculture sector (30.2% males and 67.2% females).

In the past century, the annual average temperature in Pakistan has considerably increased. By the end of the 21st century, the temperature in most parts of Pakistan is expected to increase by 0.6 to 1.0 °C [36]. Therefore, it is essential to recognize growers’ definite CC adaptation practices and their influence on maize production, particularly when Pakistan’s agricultural sector is in a transitional retro and is facing several environmental disasters. Abid et al. [37] analyzed the adaptation of maize farmers to CC and its impact on maize yield. However, they only considered an adaptation strategy under extreme weather conditions. Hence, this study could not be considered complete regarding whether the current adaptation practices of Pakistani farmers help the agricultural sector or not. This paper investigated the general adaptation approaches of growers to CC and their consistent influences on growers’ maize productivity.

The article has six sections. After the introduction, Section 2 describes the review of the literature. Section 3 presents the materials and methods. Section 4 describes the results. Section 5 presents the discussion and, finally, Section 6 outlines the conclusions and policy implications.

## 2. Review of Literature

### 2.1. Climate Change Cognition

CC cognition is a multifaceted process involving a range of psychological concepts, for instance, opinions, attitudes, knowledge, and concerns, regarding whether and how climate has been changing [38]. Cognition is affected and formed by various factors such as personal characteristics, skills, information obtained, and cultural and geographic environment [39]. Hence, efforts to measure CC perception and implement its factors are not trivial. The variation of local weather from day to day, season to season, and year to year are one of several challenges people face when trying to understand the difference between normal short-term changes and CC [40]. Moreover, short-term changes tend to be further pronounced than long-term trends and thus may have an important influence on the development of CC perceptions [41]. While some farmers whose income directly relies on the weather, such as rainfed farming, tend to perceive more accurately than their complements, they may still be unable to properly utilize their knowledge of climate variables to explain variations, sufficient to feel concerned and forced to do something about this [39]. Experiences affect perception and individuals directly influenced via risky CC measures tend to state a relatively high likelihood of such events recurring [42]. Additionally, individuals’ perceptions of CC may be influenced or adapted by the information they receive [43]. In conclusion, it should be observed that the perception part of the problem is a special phenomenon, so several individuals in a similar business may construct different CC perceptions while they experience the same weather and or climate pattern [44].

### 2.2. Association between Cognition and Adaption to CC

To defend farmers’ livelihoods that depend on agriculture, the farming sector’s adaptation to the adverse CC influences is vital [45]. In an advanced world of well-informed, well-established markets and ample opportunities and motivations, the choice to adopt or implement adaptation measures is simply a matter of assessing the net assistance of effective measures. This environment is not for smallholders and subsistence farmers in developing countries [46]. Therefore, taking adaptation measures is not an automatic or smooth process, rather, it is complicated and challenging for smallholders. For example, evidence suggests that factors such as insufficient access to insurance or credit, limited information on adaptation measures, and incomplete property rights constitute barriers to technology adoption by small and subsistence farmers [45].

Additionally, the decision to implement new technologies or production approaches frequently entails cognitive processes, such as loss aversion, mental accounting, and hyperbolic discounting, which can lead to a sub-optimal adaptation level [39,47]. This is specifically related to adaptation to CC, as even farmers with access to weather information and climate forecasts face considerable uncertainty [48]. Under these conditions, farmers’ perceptions of CC are important in understanding their adaptation decisions [49]. Adaptation entails not only an individual’s perception that something is varying or could vary, but also that they pay enough attention to this perception to be willing to act on it [50]. From this perspective e, the perception that the climate is changing can be seen as a prerequisite for adopting farming adaptation procedures [51]. The effective implementation of public policies aimed to promote adaptation measures also requires the support and contribution of the projected recipients. If they have a different view of the consequences or immediacy of CC than policymakers, implementation of the policy is likely to fail [52].

### 2.3. CC and Its Impact on Crops

Climate change directly affects agricultural production as it relies heavily on the weather and the environment, significantly reducing crop yields, and sustainable food system efforts are strongly confronted by CC [53,54]. The CC effects are obvious on rainfall, temperature patterns, weather tolerance of different crop varieties, per capita income, employment in agricultural patterns, and consequent economic activity [55]. Gmann and Horst [56] show that CC is dangerous to agriculture, water, and food, particularly in developing countries with limited natural resources. South Asian grain crops are already facing the hazardous consequences of CC. Thus, the results outline that CC negatively affects the agricultural sector and the sustainable production of crops. Numerous studies have acknowledged the inverse relationship between CC and global agricultural commodities and strengthened the sensitivity of the agricultural sector to CC. Moreover, developing countries in Asia, Africa, Latin America, and Oceania are highly concerned about CC [57,58].

Parry et al. [59] stated that the CC effects on agriculture vary by region, i.e., temperate regions may experience a positive trend in global warming (1–3 °C increase in temperature). In contrast, tropical regions can see a negative trend. Chandio et al. [60] presented that the average maximum temperature negatively influenced rice yield and the average minimum temperature facilitated rice production. Climate change impact studies on agriculture found that increasing greenhouse emissions have devastating consequences on crop yields [61]. Lu et al. [62] stated that CC might affect the quality and quantity of water which negatively contribute to food production.

Furthermore, Xie et al. [63] reported that CC is expected to reduce wheat production by 9.4% by 2050. Sosu et al. [63] explored the CC effect on grain yield. The results showed that high temperature could damage grain yield, while optimum precipitation could positively affect yield. For example, an extra 1 mm of rain could increase yields by 9 kg/ha. Ali et al. [64] studied the association between CC (through rainfall and temperature) and technological progress (through agricultural technology and fertilizers) of crops (maize, rice, and wheat) in Pakistan from 1989 to 2015. While the results showed negative impacts on temperature, rainfall, and fertilizers, modern agricultural technology had a positive impact on maize production and contributed to food security under prevailing environmental conditions.

Again, the research shows that CC influences maize production; however, local technological advancements of farmers have played an essential role in raising maize yield. Based on these outcomes, the authors recommend the design of well-positioned adaptive policies to address future climate impacts on agriculture. Zhai et al. [65] and Zhang and Yao [66] concluded that CC proved beneficial to northern and central China, but temperature influences were usually detrimental to wheat yield. In addition, south China’s rainfall, temperature, and solar radiation decreased wheat yield from 1981 to 2009 [67]. Pickerson et al. [68] used quarterly data from 1990 to 2013 to study the CC effect on China’s cereal production. The outcomes provided the long-term negative impact of CO_2_, temperature variation, and the average temperature on food production, whereas energy usage, average rainfall, arable land, and workforce positively influenced cereal production. Based on these consequences, the authors recommended the development of improved cereal varieties to counter the adverse effects of climate. This study fills this gap using these techniques and conducts empirical work to assess the CC impact on maize production in the KP province of Pakistan. Furthermore, this research can serve as a basis for other developing countries in Asia with parallel economic and climatic conditions, to address food security issues. This makes the existing survey useful not only for Pakistan but for other agriculture-based economies.

## 3. Material and Methods

### 3.1. Study Area

The research was conducted in Khyber Pakhtunkhwa (KP), a northwest frontier province of Pakistan. The province has a total area of 74,521 Km^2^ and a population of 29.531 million in 2020. The total arable land area of the province is approximately 1,643,793 hectares. The native language of the KP province is Pashto and most people belong to the Pashtun tribe. Agriculture, trade, and public services are the province’s major sources of income. The province grows several essential crops in the agricultural sector, including maize, rice, sugarcane, millet, tobacco, barley, etc. The environment of KP province varies according to its size, including most of the climatic zones in Pakistan. There is also a high degree of rainfall variation as most of the province comprises an arid ecological zone [69]. On the other hand, the eastern part of the study province is considered the wettest side of Pakistan, particularly in the monsoon period (June to mid-September) [70].

### 3.2. Sampling Approach and Data Collection

**Figure 1:**
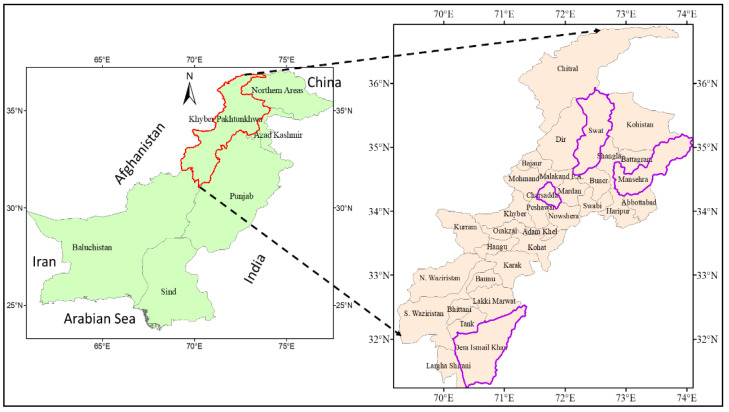
Study area map [71,72,73].

**Figure 2:**
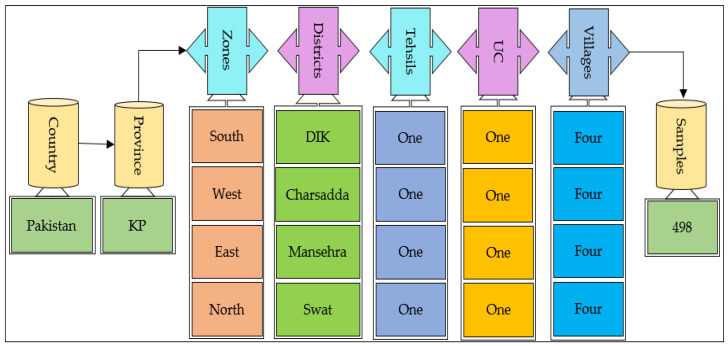
Sample distribution.

The current data were gathered in the KP province of Pakistan; 550 questionnaires were distributed to maize growers and a total of 498 questionnaires were completed. Basic information was collected from maize growers using a multi-stage random sampling method. To recognize existing farmers’ perceptions, adaptation to CC, and its impact on maize productivity in KP province, first, data were collected in four districts (Figure 1 and Figure 2), based on the contribution of agricultural output in these regions. Second, a tehsil was selected to complete the questionnaire and, third, a union council (UC) from each selected tehsil was selected. Fourth, each UC randomly tracked four villages and, finally, fundamental information was collected from maize growers in the nominated villages. The questionnaire utilized in this research was separated into multiple sections. The first portion of the planned questionnaire comprised the demographic and socioeconomic attributes of the farmers. The rest of the questionnaire was calculated to obtain info on farmers’ perceptions and adaptations to CC and its influence on maize yields. The feedback form was originally written in English and later converted into Urdu (the national language) for the convenience of respondents.

### 3.3. Modeling Adaptation to CC and Cash Crop Productivity

Di Falco et al. [74] suggested that the CC adaptation decisions and their influence on crop production could be simulated utilizing a two-stage framework [75,76,77,78]. Firstly, we utilized the selection model for CC adaptation choices. Assuming that hazard-averse growers will apply the CC adaptation approach if they generate net income, the latent variable A* can signify the net income.

A_i_* = *Z_i_a* + *η_i_*
*with A_i_* = 1, if A_i_* > 0 *and* 0 *otherwise* (1)

Farm i will select to adopt the CC adaptation strategy $\left(Ai=1\right)\;\mathrm{if}\;Ai^{*}>1\;\mathrm{and}\;0$ otherwise. The vector Z signifies *a* variable that influences growers’ adaptation decisions. Established on the observed literature on the factors of growers’ CC adaptation decisions [35,74,76,79], the characteristics of farmers and climate knowledge delivered by agricultural extension workers were selected as dependent variables for this study. Farmers’ characteristics contain gender, age, education, labor share, acreage and climate awareness. Information from the government primarily includes environmental threats for frost and drought.

Production technology simulated adaptive effects on maize productivity in the second step. The easiest way is to use ordinary least squares (OLS), which use fitness as a dummy variable in the food yield equation while using the OLS method to measure the influence of adaptation on maize production could lead to several possible difficulties. For instance, adaptation can be endogenous and, if accurate, lead to biased evaluations [31]. Furthermore, issues such as sample selection bias and inconsistency in estimation may add to and confound the outcomes [33]. An investigation by Di Falco et al. [31] shows an equation model for estimation of CC adaptation and its effect on maize productivity of endogenously transformed maize using occupied information maximum probability. The current study utilized variables related to climate perception and environment info as the selection model. Table A1 displays that climate perception and awareness substantially influenced farmers’ adaptation decisions, but not the maize production of non-adapters. Hence, they could be reflected as useful selection tools.

*Y*_1*i*_ = *β*_1_*x*_1*i*_ + *ε*_1*i*_
*if A*_*i*_ = 1 (2)

*Y*_0*i*_ = *β*_0_*x*_0*i*_ + *ε*_0*i*_
*if A*_*i*_ = 0 (3)

where $Y_{1i}\;$and $Y_{0i}$ are the maize productivity per hectare stipulated in the logarithm adopter and non-adopter, respectively. $Y_{i}$ is the input vector stipulated in the logarithm (e.g., seed, manure, labor, and fertilizers), β is the parameter vector to be estimated and ε is the error term.

Assuming that the error term in equations one and three has a three-variable normal distribution, where $\left(\eta ,\varepsilon_{1},\;\varepsilon_{0}\right)∼N\left(0,\sum \right)$ [74].

(4) $$\mathrm{COV}\;\left(\eta ,\varepsilon_{A},\varepsilon_{N}\right)=\sum =\{\begin{array}{ccc} \sigma_{n}^{2} & \sigma_{\eta A} & \sigma_{\eta N}\\ \sigma_{A\eta } & \sigma_{A}^{2} & \sigma_{AN}\\ \alpha_{m1} & \sigma_{NA} & \sigma_{N}^{2} \end{array}$$

Projected value of $\varepsilon_{1}\;\mathrm{and}\;\varepsilon_{0}$ non-zero, specified as [74,76]

(5) $$Ε\left\{\varepsilon_{1i}|A_{i}=1\right\}=\sigma_{1i}\frac{\varphi \left(Z_{i\alpha }\right)}{1-\phi \left(Z_{i\alpha }\right)}=\sigma_{1\eta }\lambda_{1i}$$

(6) $$Ε\left\{\varepsilon_{0i}|A_{i}=0\right\}=-\sigma_{0i}\frac{\varphi \left(Z_{i\alpha }\right)}{1-\phi \left(Z_{i\alpha }\right)}=\sigma_{0\eta }\lambda_{0i}$$

The endogenous switching regression (ESR) method could be utilized to examine four conditional prospects of crop productivity [74].

(7) $$E\left({𝒴}_{1i}|A_{1}=0\right)=\beta_{1\chi 1i}+\sigma_{1i}\lambda_{1i}$$

(8) $$E\left({𝒴}_{0i}|A_{1}=0\right)=\beta_{0\chi 0i}+\sigma_{0i}\lambda_{0i}$$

(9) $$E\left({𝒴}_{0i}|A_{1}=1\right)=\beta_{0\chi 1i}+\sigma_{0i}\lambda_{1i}$$

(10) $$E\left({𝒴}_{0i}|A_{1}=1\right)=\beta_{0\chi 1i}+\sigma_{0i}\lambda_{1i}$$

Equations (7) and (8) indicate the definite prospects examined in the sample. Equations (9) and (10) indicate contradictory probable results. Furthermore, the difference between Equations (7) and (9) can be utilized to quote the average treatment effects on the treated (ATT). Consequently, the variance between Equations (8) and (10) could be calculated as the average treatment effects of untreated (TU) households. For the “adopter” group, the effect of the underlying heterogeneity is the variance amid Equations (7) and (10). Likewise, for the ‘nonadopter’ set, the difference between (9) and (8) was used to measure the effect of the underlying heterogeneity. For further details on ESR models, see [74].

## 4. Results of the Study

### 4.1. Descriptive Statistics

Table 1 displays the descriptions and descriptive statistics of the investigated rural farmers. The results showed that, on average, 91.7% of farmers perceive CC, 82.7% of farmers have adopted adaptation strategies related to changes in maize planting, and 43% have obtained CC information externally. Growers had taken some steps to adapt their maize crops to CC. Generally, the key strategies included an increase in the frequency of the irrigation rate, higher fertilizers and pesticide usage, and climate-adaptive crop varieties. In addition, around 63% of growers adopted more than one adaptation approach and 2% adopted more than three adaptation approaches. Generally, growers were aware of higher temperatures and lower rainfall at the study sites.

**Figure 3:**
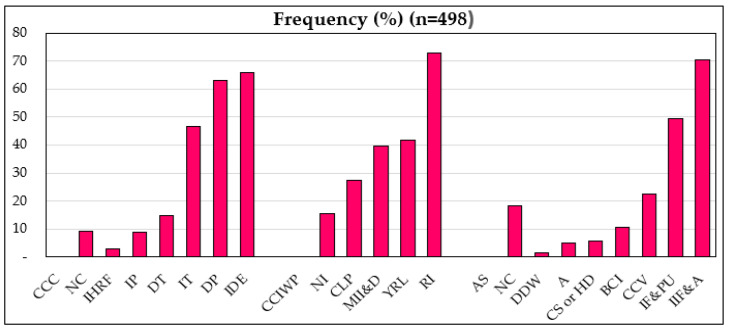
Percentage of farmers’ perceptions of CC and its influence on maize productivity and farmers’ adaptation practices. Note: Climate change cognition (CCC) = No change (NC), Increased heavy rainfall (flood) (IHRF), Increased precipitation (IP), Decreased temperature (DT), Increased temperature (IT), Decreased precipitation (DP), Increased drought event (IDE). Climate change impact on maize production (CCIMP) = No influence (NI), Crop loss due to precocity (CLP), More infestation of insects and diseases (MII&D), Yield reduction due to lodging (YRL), Require more irrigation (RI). Adaptation strategies (AS) **=** No change (NC), Drill the deep well (DDW), Afforestation (A), Change seeding or harvesting date (CS or HD), Buy climate insurance (BCI), Change crop varieties (drought tolerant and disease) (CCV), Boost fertilizer and pesticides usage (BF&PU), Rise irrigation frequency and amount (IIF&A).

Furthermore, we assembled comprehensive production information at different production levels. Labour effort is categorized by family labor and service. The average maize crop sowing area was 0.771 hectares and the maize production was 1987 kg/hectare. The main inputs for farmers were fertilizers, housework, and machinery, with low rent and labor costs. The average age of respondents was 56 and 60% of them had more than 9 years of schooling. Detailed information on farmers’ observations of CC, the influence of CC on maize yield, and farmers’ adaptation practices are presented in Figure 3.

In this study, farmers who selected as a minimum one adaptation approach were named “adopters” and those who did not take any approach were named “non-adopters”. Table 2 reveals the variances in family attributes between adopters and non-adopters; the average maize crop yields were substantially higher for non-adopters than adopters. It was apparent that some contributions by non-adopters, for instance, employment costs and irrigation costs, were substantially higher when compared with adopters. However, adopters were more aware of CC and its influence on maize crop production, as well as of access to CC information.

### 4.2. The CC Adaptation Measurement and Maize Productivity Equation

The ESR model estimates adaptive choices and mutually produces the subsequent equation [76]. Table 3 displays the outcome of the ESR model and the second column displays the assessed outcomes of the adaptation selection equation, indicating the CC adaptation determinants. The area coefficient was optimistic and statistically substantial, indicating that the farmers with more planting areas were more likely to adopt CC adaptation approaches. The impact of CC perception and climate info was both optimistic and statistically substantial, signifying that maize growers who knew about CC and had access to it were more likely to adapt to CC. The estimated values in columns 3 and 4 of Table 3 illustrated the ESR of the maize crop production. The assessed coefficient of a correlation coefficient $r_{0}\;\mathrm{or}\;r_{1}$ was not significantly different from zero, suggesting that sample selection bias may not exist in the study sample [74]. However, variances in the coefficients of the maize productivity equation between adopters and non-adopters suggested that the samples were heterogeneous [74,80]. The outcomes in Table 3 specify that area is the key factor in clarifying lower maize productivity in both adopter and non-adopter groups. Nevertheless, the gender of the respondents, schooling, farmyard manure, housework, irrigation system, and rent appeared to exert varying influences on maize yields for adopters and non-adopters. The outcomes in the three columns showed that schooling and irrigation systems were substantial optimistic factors for adopters’ maize production. Furthermore, household labor input appeared to harm maize crop yields for non-adopters.

Table 4 displays farmers’ maize production expectations under actual and counterfactual conditions, along with estimates of average treatment effects and underlying heterogeneity effects. Units (1) and (2) signify the expected maize production observed in the samples. Unit (3) denotes the expected maize production when the adopter decides not to adapt and unit (4) the probable maize production when the non-adopter agrees to adopt. If they do not adapt, adopters will increase production by 955 kg/ha (28%). Likewise, if they adapt, non-adopters will have reduced production by 519 kg/ha (13%). Furthermore, the last row of Table 4 displays that in the counterfactual case, the production of adopters will be substantially higher than that of non-adopters. The substantial heterogeneity influence means that irrespective of the CC problem, adopters are “well producers” than non-adopters because of a few significant sources of heterogeneity.

## 5. Discussion

Previous studies have reported maladaptive outcomes of various adaptation actions in the agricultural sector [21,81,82]. Why did adaptation procedures fail to reduce climate hazards and have adverse outcomes in these studies? Here are the main explanations for why major adaptation actions have failed. Firstly, in the study reported by Liu et al. [83], during the grain filling period of crops, the quantity and frequency of irrigation would be lessened appropriately [83]. Hence, adaptive actions by farmers to enhance irrigation frequency and volume due to decreased rainfall could negatively affect maize production if increased irrigation is performed at inappropriate times. Secondly, fertilizer inputs in Ethiopia and Nepal are important in increasing grain yields [74,76]. However, Pakistan is using a higher rate of inorganic fertilizers compared to Ethiopia and Nepal [84]. Various empirical studies suggest that smallholder farmers are at high risk and are willing to use additional fertilizers to evade the adverse effect of probable climate hazards on agricultural production [21,85,86,87]. Nevertheless, due to the limited technical knowledge, non-availability of agricultural labor, and frequent use of old traditional experiences or habits, the excessive application of chemical fertilizers and pesticides by local farmers are continuously being practiced in Pakistan, resulting in severe environmental hazards [12]. Excessive chemical fertilizers may reduce the arable land fertility, cause water pollution [12,84,85] and erode supportable agricultural advancement [12,86]. Consequently, adaptive actions to upsurge fertilizer usage in response to CC risk increase grain yields when soil fertility is poor. However, excessive use of fertilizer and pesticides by farmers may negatively influence maize productivity and disrupt ecosystem stability. Thirdly, some farmers have switched crop cultivars to drought and disease-resistant maize cultivars in response to reduced rainfall and increased pests and diseases. Nevertheless, drought and disease-resistant maize cultivars may not be high-yielding and adapting new cultivars to complex ecological conditions may lead to crop failure.

Adaptation is crucial for reducing the havoc of CC, maintaining growers’ incomes, and ensuring sustainable agricultural growth [88]. The agricultural sector in Pakistan is facing severe resource and ecological limitations such as irrigation scarcity and ecosystem degradation, though self-adaptation by smallholders may not be feasible. Therefore, local governments of KP should take essential measures for rural farmers to implement suitable and appropriate adaptation approaches, as the government oversees the agricultural infrastructure construction of the “water and irrigation conservancy arrangement, agricultural information and farming product quality supervising system” and agricultural discipline and equipment advancement. Subsequently, small rural farmers could waste their energy and resources or even suffer losses if they are alone. According to our findings, on the one hand, for the farmers’ benefit and sustainable agricultural development, there is an urgent need to contrivance technical irrigation and fertilization strategies to improve fertilizer and water efficiency.

Furthermore, it is necessary to focus on the study and growth of seed varieties with better germplasm, thereby contributing to abiotic or biotic stress resistance and improved maize production under adverse environmental conditions. Moreover, the government should ensure the easy and safe transfer of scientific knowledge, precautionary and usage guidelines, and materials to the local farmers to achieve the best possible outcomes under the prevailing climatic conditions. This study investigated the rural farmers’ perception, adaptation to CC, and its influence on maize productivity. The explanations behind this marvel and whether it signifies a general situation across several areas or crop varieties will be further investigated.

## 6. Conclusions, Policy Implications, and Limitations

The CC is a global environmental risk for all economic sectors, especially agriculture. In Pakistan, the global and regional climate dynamics have severely affected agricultural productivity and rural livelihoods for decades. Potential losses at the farmhouse level can be reduced by timely adaptation to CC. Pakistan faces risky weather events such as untimely torrential rainfalls and flash floods that cause massive damage to the crops and property of farmers. The losses mentioned above are expected to rise with the increasing impact of CC in the coming years. Given the agriculture sector’s importance to national economies and rural livelihoods, the significance of CC adaptation methods is profound. While adaptation stratagems are critical, not all smallholders use them. Most small rural growers and associated urban populations in emerging nations, including Pakistan, are massively reliant on the agriculture sector. Therefore, acclimatization to the drastic impacts of CC may be dangerous to encouraging national food security and protecting rural household livelihoods. The primary purpose of this paper is to discuss farmers’ autonomous CC adaptation approaches and their impact on maize crop production. Based on a study of 498 farmers in rural Pakistan, this study found that more than 80% of farmers knew about CC and more than 70% of farmers adopted adaptive strategies on their own. Farmers’ arable land area, CC awareness, and information mainly determine their adaptation verdicts. In addition, growers have limited adaptation guidelines, primarily causing increased irrigation and additional fertilizers and pesticide usage. Results showed that farmers’ CC adaptation policies extensively improved maize productivity, signifying that there might be maladaptive behaviors in farmers’ CC adaptation. Overall, CC adversely affected the yields of major food crops, such as maize, in Pakistan. With a large population living below the poverty line and a rapidly growing population, the country is about to face food security problems. The government must develop a sustainable approach to address this challenge to safeguard the commonalities of food security.

CC adaptation is expected to see an upsurge in maize yields. Access to adequate resources and adaptive info measures could be utilized as short-term policies that can sustain crop production. In terms of adaptation benefits, a combination of multiple adaptation measures is superior to a single adaptation measure. Adaptation using CC potential levels not only supports farmers’ net financial position and improved livelihoods but also increases maize crop productivity at the national level. To correctly exploit adaptation assistance, region-specific guidelines must be established based on region-specific farmer needs and climate risks. Increased productivity is a huge benefit of using adaptation measures, as demonstrated by the research’s empirical findings. Insufficient adaptation procedures and improvements and inadequate information are some of the key limitations in enjoying the latent benefits of adaptation. Adaptation limitations can be appropriately addressed via easy access to CC information, raising awareness of adaptation measures, educating farmers, and building their capacity through the collaboration and active participation of governments and NGOs. Since smallholders have a mass presence in many farming communities in Pakistan, special attention needs to be paid to appropriate policy measures to address the resource constraints of smallholders. While precise local CC adaptation by agriculture is required, investigation and assets are also required at macro levels, such as resource endowments, commodity costs, and the environmental impacts of international and regional development. All these improved CC adaptation measures involve policy measures that have significant long-term impacts on agricultural productivity.

## Figures and Tables

**Table 1:** Variable names, definitions, and descriptive statistics for the sample.

Variable Name	Explanation	Mean (S.D)
Adaptation	Dummy = 1, if the farmers adapted to CC, 0 otherwise	0.827 (0.377)
Maize productivity	Maize productivity (kg/ha)	1987.11 (451.08)
Farm area	Farm area under maize (hectare)	0.771 (1.992)
Maize seeds	Seeds usage per hectare kg/ha	1129.651 (364.152)
Agrochemical fertilizer	Agrochemical fertilizers usage per hectare (PKR)	2476.440 (697.026)
Farm manure	Farm manure usage per hectare (PKR)	171.858 (564.062)
Pesticide	Pesticides usage per hectare (PKR)	542.944 (296.527)
Household labor	Household labor input per hectare (PKR)	2638.080 (2135.371)
Employment cost	Employment expenditure per hectare (PKR)	180.419 (581.991)
Technology	Technology charge per hectare (PKR)	1526.853 (701.715)
Irrigation charges	Irrigation charge per hectare (PKR)	463.738 (459.876)
Rental	Rental expenditure per hectare kg/ha	32.684 (94.295)
Gender	Dummy = 1, if a farmer is male, 0 otherwise	0.723 (0.448)
Age	Farmers’ age	56.131 (11.319)
Educational status	Dummy = 1 if farmer has an education, 0 otherwise	0.615 (0.487)
Household size	Number of household size	0.465 (0.124)
Workforce share	Workforce as a share of the total household population	0.604 (0.221)
Agr-Extension service	Dummy = 1 if the farmers access service, 0 otherwise	0.451 (0392)
Climate cognition	Dummy = 1 if the farmers believe that CC, 0 otherwise	0.917 (0.276)
CC impact on maize productivity	Dummy = 1 if farmers believe CC affects maize productivity, 0 otherwise	0.857 (0.351)
Climate Information	Dummy = 1 if farmers obtained warning climate info, 0 otherwise	0.430 (0.496)

**Table 2:** Farmstead and farmers’ attributes of adopters and non-adopters.

Variable	Adopters	Non-Adopters	Difference
	Mean (S.D)	Mean (S.D)	
Adaptation 1/0	1.000 (0.000)	0.000 (0.000)	
Maize productivity	1811.636 (303.471)	1685.140 (457.803)	126.496 **
Farm area	0.809 (2.146)	0.588 (0.948)	0.221
Maize seeds	1139.629 (306.923)	1127.578 (375.437)	12.051
Agrochemical fertilizer	544.795 (300.001)	534.028 (281.712)	10.767
Farm manure	304.387 (727.207)	144.332 (521.404)	160.055
Pesticide	2520.898 (721.167)	2467.206 (692.978)	53.692
Household labor	2708.825 (2219.873)	2297.456 (1644.566)	411.369
Employment cost	408.226 (905.401)	133.105 (478.036)	275.121 **
Technology	1547.522 (651.598)	1427.338 (906.063)	120.184
Irrigation charges	593.72 (469.75)	436.741 (454.060)	156.979 **
Rental	33.584 (96.138)	28.349 (85.563)	5.235
Gender	0.731 (0.444)	0.685 (0.469)	0.046
Age	56.238 (10.256)	55.574 (11.241)	0.664
Educational status	0.612 (0.488)	0.63 (0.487)	−0.018
Household size	0.175 (0.127)	0.175 (0.127)	0.038
Workforce share	0.597 (0.218)	0.637 (0.236)	−0.040
Agr-Extension service	0.621 (0.495)	0.64 (0.491)	−0.019
Climate perception	0.977 (0.150)	0.63 (0.487)	0.347 ***
Climate influence on maize	0.977 (0.150)	0.278 (0.452)	0.699 ***
Climate information	0.508 (0.501)	0.056 (0.231)	0.452 ***

Note: ** and *** denote statistical significance of 5% and 1%, respectively.

**Table 3:** Regression results from endogenous switching of CC adaptation and influences maize productivity.

Variable	Adaptation	Maize Yield (Log)	
		Adopters	Non-Adopters
Gender	0.263 (1.10)	−0.003 (−0.07)	0.118 ** (2.55)
Age	−0.002 (−0.20)	0.001 (0.58)	0.000 (0.06)
Educational status	−0.017 (−0.07)	0.065 * (1.91)	0.060 (1.15)
Household size	0.029 (0.062)	0.022 (0.027)	0.005 (0.023)
Workforce share	−0.616 (−1.36)	0.070 (0.98)	0.138 (1.48)
Agr-Extension service	0.028 (0.063)	0.021 (0.026)	0.005 (00.023)
Farm area	0.298 * (1.85)	−0.021 ** (−2.55)	−0.073 ** (−2.17)
Maize seeds (log)	-	−0.053 (−1.28)	−0.098 (−0.85)
Farm manure (log)	-	−0.002 (−0.69)	0.006 * (1.78)
Agrochemical fertilizers	-	0.068 (1.26)	0.045 (0.64)
Pesticide (log)	-	0.042 (1.57)	0.051 (1.27)
labor (log)	-	−0.009 (−1.19)	−0.105 *** (−2.97)
Employment cost (log)	-	−0.007 (−1.35)	−0.001 (−0.10)
Irrigation charges (log)	-	0.012 *** (4.88)	−0.002 (−0.27)
Technology (log)	-	−0.007 (−1.10)	−0.006 (−1.13)
Rental (log)	-	−0.009 ** (−2.26)	−0.009 (−1.39)
Rent (0/1)	0.157 (0.43)	-	-
Climate perception	1.877 *** (4.91)	-	-
Climate information	1.259 *** (4.65)	-	-
Constant	−0.923 (−1.34)	8.189 *** (16.63)	9.613 *** (9.81)
σ1	-	−1.402 *** (−29.70)	
σ0	-		−1.999 *** (−10.83)
p1	-	0.347 (1.54)	
p0	Adaptation		0.584 (0.70)

Note: *, **, *** signify statistical significance at 10%, 5% and 1%, respectively; the *t*-value in parentheses.

**Table 4:** Influences of adaptation on projected average maize crop productivity; Treatment and Heterogeneity Effects.

Sub-Samples	Decision Stage		Treatment Effects
	Adaptation	Non-Adaptation	
Adopters	(1) 1387.93 (12.302)	(3) 1815.832 (15.010)	TT= −427.902 *** [−2.799]
Non-adopters	(4) 1541.783 (22.339)	(2) 1801.726 (27.524)	TU= −259.943 *** [−5.185]
Heterogeneity influences	BH_I_ = 192.293 *** [1.885]	BH_2_ = 628.213 *** [1.739]	TH = −435.92 [−5.353]

Note: The standard error is in brackets and the *t* value is in square brackets, *** represents 1% statistical significance. TT: the effect of the treatment (i.e., adaptation) on the treated (i.e., farm households that adapted); TU: the effect of the treatment (i.e., adaptation) on the untreated (i.e., farm households that did not adapt); BH*_i_*: the effect of base heterogeneity for farm households that adapted (i = 1) and did not adapt (i = 2); TH = (TT–TU), i.e., transitional heterogeneity.

## Appendix A

**Table A1:** Parameter estimates—Validity testing of selection tools.

Variables	Probit Model ^a^		OLS Model ^b^	
	Adaptation 1/0		Non-Adopters Productivity per Hectare	
Gender	0.245	0.237	0.116 **	2.09
Age	–0.003	0.0011	0.000120	0.05
Educational status	0.033	0.0223	0.089	1.44
Workforce share	–0.448	0.0344	0.144	1.31
Agri-Extension services	0.444	0.110	0.207	1.48
Farm area	0.015	0.010	–0.059	–1.38
Climate cognition	1.878 ***	0.0333	0.011	0.056
Climate information	1.229 ***	0.262	–0.136	0.0109
Maize seed (log)	-	-	–0.029	–0.30
Pesticides (log)	-	-	0.054	1.14
Agrochemical fertilizers (log)	-	-	0.034	0.40
Technology (log)	-	-	–0.0138	–0.22
Irrigation (log)	-	-	0.00104	0.12
Labor effort (log)	-	-	–0.084 *	–2.01
Employ expenditure (log)	-	-	0.00365	0.52
Rental (log)	-	-	–0.078	–1.19
Rent (0/1)	0.138	0.335	-	-
Constant	–0.750	0.603	7.816***	9.16
Wald test on information sources	*χ*^2^ = 77.55 ***		F-stat. = 1.76	
Sample size	498		53	

Note: Model ^a^ = (pseudo R^2^ = 0304); Model ^b^ = (R^2^ = 0.445). *, ** and *** signify statistical significance at 10%, 5% and 1%, correspondingly.
